# Potentialities and limitations of Interprofessional Education during graduation: a systematic review and thematic synthesis of qualitative studies

**DOI:** 10.1186/s12909-023-04211-6

**Published:** 2023-04-12

**Authors:** Jéssica Rodrigues da Silva Noll Gonçalves, Rodrigo Noll Gonçalves, Saulo Vinicius da Rosa, Juliana Schaia Rocha Orsi, Karoline Maria Santos de Paula, Samuel Jorge Moysés, Renata Iani Werneck

**Affiliations:** 1 School of Life Sciences, Pontifical Catholic University of Paraná, No. 1155, Imaculada Conceição Street, 80215-901 Curitiba, Paraná, Brazil; 2 Postgraduate Programme in Public Policy at the Federal University of Paraná, No. 632, Prefeito Lothário Meissner Avenue, Curitiba, Paraná, 80210-170 Brazil

**Keywords:** Interprofessional education, Students, Education, Professional, Interdisciplinary communication

## Abstract

**Background:**

Rapid demographic, epidemiological, technological, cultural/behavioural, and educational transitions, as they become more complex, demand new integrated and complementary professional skills and abilities. Interprofessional Education (IPE) is a promising alternative to deal with these changes, especially in courses in the health area. This systematic review was to explore the potentialities and limitations of IPE, from the perspective of undergraduate students, through a thematic synthesis of qualitative studies.

**Methods:**

A thematic synthesis of qualitative studies was conducted. The question elaborated for this review was: “What is the impact of interprofessional education on the teaching and learning of students in the health area inserted in Higher Education Institutions?”. The search strategy was performed in the electronic databases PubMed, Latin American and Caribbean Literature in Health Sciences (LILACS), Cochrane Library, and Scientific Electronic Library Online (SciELO). In addition, searches were carried out in grey literature on the ERIC platforms, ProQuest Disserts and Theses, and Academic Google. The assessment of the quality of the studies was carried out using the Critical Appraisal Skills Programme tool. Data were summarized through thematic synthesis. From the databases, 8,793 studies were identified. After standardized filters procedures, critical summaries, and assessment of relevance to the eligibility criteria, 14 articles were included.

**Results:**

The synthesis of the studies revealed the potential of this teaching approach, arranged in three analytical themes: learning from each other and about them; the value of education and interprofessional practice; patient-centred health care. On the other hand, some limitations were also identified, such as barriers related to EIP; the difficulties related to teaching methodologies.

**Conclusion:**

Overcoming the identified limitations can enhance the results of the IPE, in view of its impact on the education of students and on the health care of the population.

**Supplementary Information:**

The online version contains supplementary material available at 10.1186/s12909-023-04211-6.

## Introduction

Recent global changes, combined with the great transitions that human societies are experiencing, increasingly require the training of university professionals with critical thinking and the ability to work in interprofessional teams, to act in the face of new challenges, especially in the health area [1–3]. However, in most Higher Education Institutions (HEIs), classes are still concentrated in departmentalized spaces (or educational and professional “silos”), restricting the possibilities for students to learn and interact with other courses and professions [4]. Nevertheless, the commission entitled “The independent Lancet Commission” [5] underscored the need for an international effort to transcend professional boundaries that persist into the twenty-first century.

Interprofessional Education (IPE) constitutes a promising alternative to deal with these new global challenges, providing students with opportunities for mutual learning with colleagues from other courses and professions, aiming at the development of new skills and abilities for effective future work in an interprofessional team [6–9].

In 2010, the World Health Organization (WHO) published the “Framework for Action on Interprofessional Education & Collaborative Practice”, marking the IPE as “[…] an innovative strategy that will play an important role in reducing the global crisis in health workforce” [1]. This interaction, carried out in a coordinated and collaborative manner, between different courses covered by the health area, is an essential component to improve the provision of health care [10, 11].

IPE transcends siloed teaching and learning approaches by emphasizing integrated learning and mutual respect between different professions in response to the new demands of health systems [12–14]. However, Thistlethwaite [15] warns that just bringing together students from different courses is not enough. HEIs should institute the use of prepositions “on, with and among themselves” for an authentic interprofessional learning experience. That way, for the team to develop interprofessional teamwork, it is necessary to understand the processes that permeate this teaching approach in a participatory and intersectional way [16].

Based on such suppositions, the IPE seems to be an auspicious alternative in the formation of competent students to act in the face of the new complexities demanded by the rapid global transitions [13, 14]. Thus, it is relevant to verify the impact of this approach and assess whether it really offers a better educational experience for those involved, streamlining, and overcoming the traditional curricula, rather than just duplicating them [17]. For this, it is essential to understand the perceptions of the inserted students in its different learning contexts [18].

The qualitative approach is not a consensus method, but a context method. In this way, it presents variation, structure, and ability to lend itself to different environments. This is what makes it suppositional rather than propositional [19]. Thus, qualitative research is a viable alternative to unveil the students’ understanding of IPE, since, according to de Souza Minayo [20], this approach “works with the universe of meanings, motives, aspirations, beliefs, values and attitudes, the which corresponds to a deeper space of relationships, processes and phenomena”. Therefore, the objective of this systematic review was to explore the potentialities and limitations of the IPE, from the perspective of undergraduate students in the health area, through the thematic synthesis of qualitative studies and mixed methods.

## Methods

This systematic review is a qualitative study and was conducted in accordance with the guidelines in the Preferred Reporting Items for Systematic Review and Meta-Analysis PRISMA [21] statement. The qualitative approach was used to complement the findings of the quantitative review published by the own authors [12]. In this way, some sections of the method may be like the structure of the quantitative study.

Qualitative reviews are based on the analysis of human experiences, cultural and social phenomena. Thus, they focus on involvement between the participant and the intervention. Research using this approach should conduct questions centred on the perspective of individuals who experienced a certain phenomenon. In this sense, the evolution of methods for conducting systematic reviews has advanced thinking about the types of questions needed to answer and provide the best evidence-based care. Thus, we chose to use the PICo mnemonic proposed by Munn et al. [22].

The question elaborated for the review was: “What is the impact of interprofessional education on the teaching and learning of students in the health area inserted in Higher Education Institutions?”.

### Population (P)

Pre-registration and undergraduate health course students.

### Phenomena of interest (I)

Potentialities and limitations.

### Context (Co)

Interprofessional education.

Potentialities relate to factors that contributed to increasing student readiness for the IPE, while limitations were the reasons that contributed to reduction of readiness for this teaching and learning approach from the perspective of students.

### Eligibility Criteria

Studies that addressed the potentialities and limitations of interprofessional education from the perspective of undergraduate students; qualitative analysis; mixed studies (when it was possible to extract individualized data from the qualitative part); without the restriction of publication date and language. Research whose data were not primary were excluded; studies in which students’ perception of IPE was not the main objective; studies that identified the perception of professors, patients, mentors, and graduate students; editorial letters, pilot studies, case reports, simulations, virtual experiences, workshops, guidelines, and research reports.

### Selection and data collection

To locate the terms and search strategy, the Medical Subject Headings (MeSH) and Health Sciences (DeCS) descriptors were consulted. The search strategy was carried out in the electronic databases: PubMed, Latin American and Caribbean Literature in Health Sciences (LILACS), Cochrane Library and Scientific Electronic Library Online (SciELO), between August 4 and 5, 2020. In addition, searches were performed in grey literature on ERIC platforms (ProQuest), dissertations and theses using the database ProQuest Disserts and Theses, as well as Google Scholar. The reference list of all included primary studies was searched. The search strategy terms were adapted to suit the rules of each database (Supplement 1).

The complete articles, which could not be obtained by these means, were requested from direct contact with the respective authors.

**Fig. 1:**
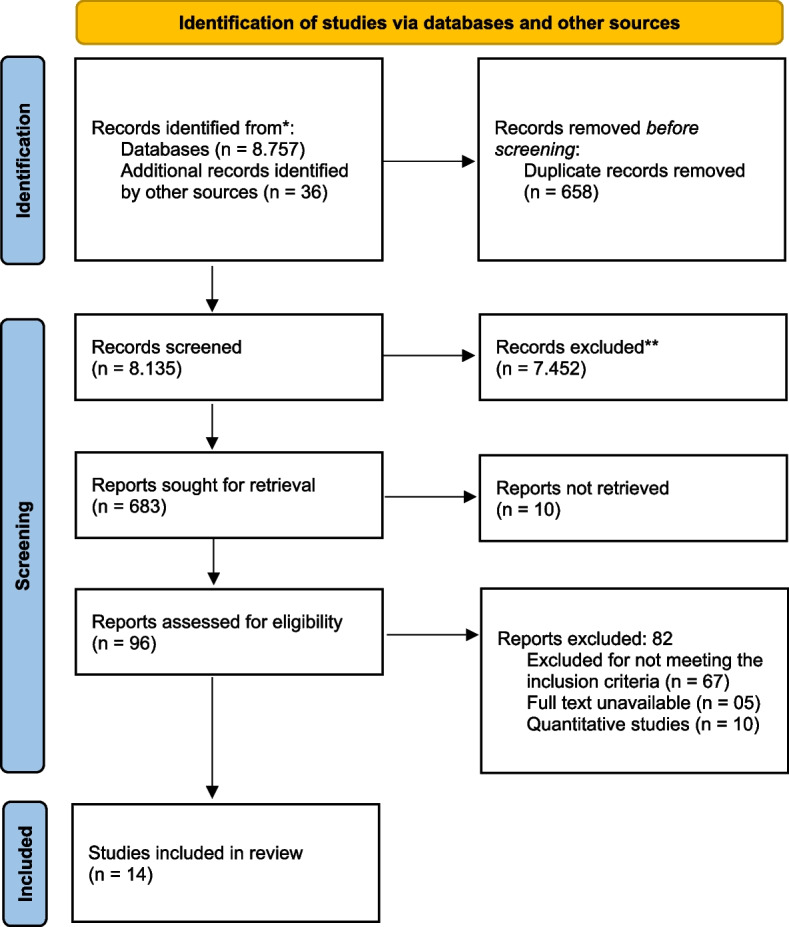
Flow diagram of study

After consulting the databases, the studies from the search strategy were imported into the EndNote X8.2 software. Duplicate studies were removed, following an initial selection of titles and abstracts by two independent reviewers (J.R.S.N.G. and R.N.G.), considering the inclusion and exclusion criteria. Subsequently, the full texts of the other studies were obtained for analysis and confirmation of the eligibility criteria, with subsequent data extraction. The discrepancies found at the end of each process were discussed with a third reviewer (S.V.R.) to reach a consensus. At this stage, the agreement between reviewers using the Cohen Kappa statistical method was 77% and the complied flowchart is described in Fig. 1.

### Critical evaluation of included studies

The quality assessment of the studies included in the review was performed using the instrument to assess the quality of qualitative research Critical Appraisal Skills Programme (CASP) [23]. The checklist consists of 10 questions (Supplement 2), consisting of one or more questions that assess items regarding the selection criteria of the studies; research plan; data collection and analysis; ethic; reflexivity; and implications of qualitative research. According to the guidelines for using this instrument, the first three questions are essential. Thus, if in any of the essential questions the answer is “no”, the article should be excluded, as it is outside the minimum criteria of the methodological standards.

The CASP was applied independently by two reviewers (J.R.S.N.G. and S.V.R.), with disagreements being resolved a posteriori, after discussion and consensus. The articles received different scores for each criterion, being: 1 – when the criterion was fulfilled; 0.5 – when the criterion was partially fulfilled; 0 – when the criterion was not fulfilled. In this logic, the maximum score that could be obtained for each article would be 10 points [24]. The agreement between reviewers, in this new stage, using the Cohen’s Kappa method was 74%.

### Data extraction and analysis

For the extraction, a form was elaborated containing the following information: title of the study; authors/year of publication; country; study design; educational intervention; course’ frequency; educational strategies; duration of the course; school year; courses involved in IPE; assessment instruments; evaluation method; sample size; sex; age of students; data collect; data analysis; potentialities and limitations of IPE; limitations of the study; observations; and quality score (CASP). This step was performed independently by one of the reviewers (J.R.S.N.G.) and, subsequently, checked by another reviewer (R.N.G.).

To provide a synthesis of the findings of the studies included in the systematic review, a diagram was created to illustrate the frequency and relevance of interpretive codes, through the PowerPoint software for Mac (version 16.38). The geometric figures are equivalent to the interpretive codes – the circles represent the potentialities and the squares the limitations of the IPE – and the colours represent the analytical themes. Their sizes can vary according to the number of references about a given code. The superimposition of the figures highlights the interrelationship between codes [24].

To explore the potentialities and limitations of the IPE, a synthesis of qualitative studies was performed by two reviewers (J.R.S.N.G. and S.V.R.). For this purpose, the reference from the Thematic Synthesis by Thomas and Harden [25] was used. This method was developed and applied in systematic reviews to address issues relating to the personal perspectives and experiences of those involved.

**Fig. 2:**
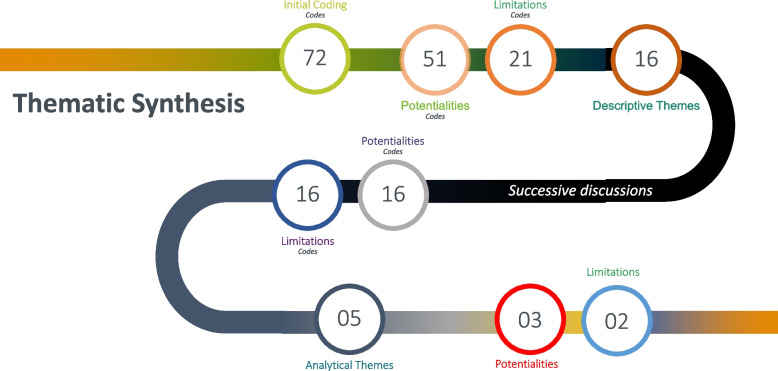
Illustration coding of thematic synthesis

**Fig. 3:**
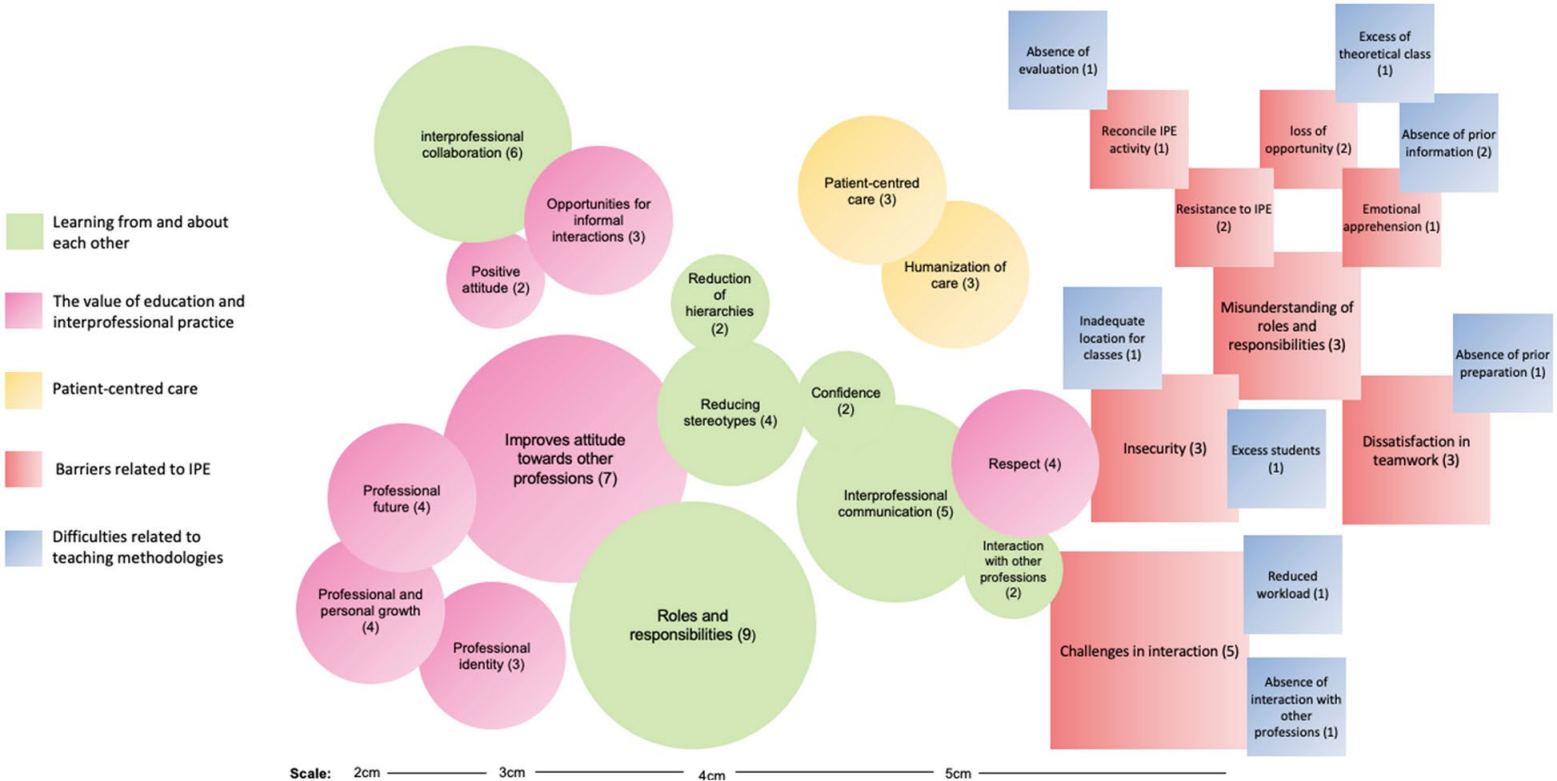
Analytical themes and interpretive codes regarding the potentialities/limitations of IPE, based on the original studies. Curitiba, 2021 Note: Size of circles and squares (code density indication): 1–2 references = 2 cm; 3–4 references = 3 cm; 5–6 references = 4 cm; and above 6 references = 5 cm. The colours of the circles/squares represent the analytical themes. The potentialities (circles) of the IPE are factors that led students to a positive perception of the student towards the IPE, and the limitations (square) are those that led to a negative perception

In the first stage, the results of each study were selected and coded manually, line by line (free codes), independently by two reviewers (J.R.S.N.G. and S.V.R.). The original codes, cited in the articles, and the additional codes, identified by the reviewers, were included in the analysis. In the second stage, the free codes were organized into initial “descriptive themes”, based on the similarities and differences found is described in Fig. 2. These themes were defined through discussion among reviewers (Fig. 3, inserted in the summary of results section). The third stage involved the development of “analytical themes”, through new interpretive constructions that evolutionarily synthesize, that is, with new meanings, the findings of all the included studies.

## Results

The process of processing and selecting the included articles is described in Fig. 1, already presented. Reiterating, after the duplicate removal step, 8,135 titles remained; 96 texts were classified as per to explained criteria, for the full reading step; 14 references were included. The main reasons for the exclusions were: obviously, not meeting the inclusion criteria (*n* = 67); full text unavailable (*n* = 5); and quantitative studies (*n* = 10).

### Characteristics of included studies

Table 1 presents a summary of the characteristics of the included studies. They were published between the years 2004 and 2020, being mainly from the USA [26–29] and the UK [30–32]. Among the included studies, three did not report the periodicity of IPE activities [33–35]. However, among those who cited, four were related to weekly and monthly activities [18, 26, 31, 36], and nine studies encompassed three or more professions [27–29, 32–37]. Regarding the courses involved in EIP activities, the following stood out: medicine, nursing, pharmacy, and physiotherapy.

**Table 1:** Categorization and characteristics of included studies (results of individual studies may belong to more than one category). Curitiba, 2021

Category	n	References
**Courses (*N* = 58)**		
* Medicine*	09	[18, 26–28, 30, 33–36]
* Nursing*	08	[27, 28, 31–35, 37]
* Pharmacy*	07	[27–30, 32–34]
* Physiotherapy*	05	[29, 32, 34, 36, 37]
* Social Work*	03	[32, 33, 37]
* Dentistry*	03	[27, 28, 34]
* Nutrition and Dietetics*	03	[27, 32, 36]
* Occupational Therapy*	03	[29, 31, 37]
* Biomedical Laboratory Sciences*	02	[34, 37]
* Radiography*	02	[32, 37]
* Public Health*	02	[27, 28]
*Other courses (with only one quote each)	11	[27, 28, 32, 35]
**Number of courses involved**		
* A single profession*	02	[18, 26]
* Two professions*	02	[30, 31]
* Three professions or more professions*	09	[27–29, 32–37]
**Frequency**		
< *one week*	02	[28, 30]
* Weeks to months*	04	[18, 26, 31, 36]
* Semesters*	01	[37]
* Years*	03	[27, 29, 32]
* Not reported (NR)*	03	[33–35]

*other courses: Health Sciences; Foundation Paramedics; Paramedic Science; Radiation Therapy; Social Education; Medical Laboratory Science; Health Professions; Diagnostic Radiography; Midwifery; Health Sciences; Allied Healthcare

The methodological design of three articles was mixed methods [29, 34, 35] and ten of qualitative synthesis [18, 26–28, 30–33, 36, 37]. In addition, eight studies used the focus group as a form of data collection [27, 28, 31–35, 37] (Table 2).

**Table 2:** Summary of characteristics of the studies included in this systematic review. Curitiba, 2021

Author [ref]	Country	Study design	Educational intervention	Duration	Academic year	Courses	Data collection/Analysis	Sample size	Gender	Age range
**Telford e Senior **[31]	UK	Qualitative research design	Module on public health in contemporary practice	3 weeks	NR	Occupational therapy and nursing	Focus groups and semi-structured interview guide/ Thematic analysis	12	NR	20 to 45 years
**Shelvey, Coulman and John **[30]	UK	Qualitative research design	An IPE session focused on therapeutics and prescribing	2-h session	3rd year medical and 3rd or 4th year pharmacy students	Medicine and pharmacy	Semi-structured one-to-one interviews/ Thematic analysis	18	Gender (11 female and 07 male)	NR
**Haugland, Brenna and Aanes **[37]	Norway	Qualitative research design	Three interprofessional modules	3 semesters	3rd year	Nursing, radiography, occupational therapy, physiotherapy, social education, social work, and biomedical laboratory sciences	Focus groups/ Systematic text condensation	31	Gender (2 male and 29 female)	20 to 48 years
**Odole, Odunaiya and Ajadi **[34]	Nigeria	Mixed method study	Non-clinical and clinical phases	NR	NR	Medical laboratory science, dentistry, medicine, nursing, pharmacy, and physiotherapy	Focus Group/ Thematic analysis	13	NR	NR
**Thompson et al. **[18]	Canada	Qualitative research design	Case-based learning in small groups and didactic lectures	3 years	1st to 3rd years	Medicine	Questionnaire/ Content analysis	47	NR	NR
**Darlow et al. **[36]	New Zealand	Qualitative research design	Long-term disease management program in primary care	11-h IPE program	3^rd^ to 5th year	Dietetics, medicine, physiotherapy, and radiation therapy	Nominal Group Technique/ Thematic analysis	40	Gender (30 female and 10 male)	19 to 29 years
**Ding et al. **[26]	USA	Qualitative research design	Interprofessional collaborative practice in General Medicine teaching services	4-week	3rd to 4th year	Medicine	Semi-structured interviews/ Deductive and inductive qualitative analysis	24	NR	NR
**Estrada et al. **[27]	USA	Qualitative research design	Mandatory course for students in the health area and the Institute of Food and Agriculture Sciences	1 year	1st year	Medicine, dentistry, nursing, pharmacy, nutrition, public health, and health professions	Focus groups/ Inductive analysis	28	Gender (24 female and 04 male)	NR
**Johnson e Howell **[28]	USA	Qualitative research design	International apprenticeship program—health brigades	5 days	1st to 3rd year	Dentistry, health sciences, medicine, nursing, pharmacy, and public health	Observation, focus group, interview and written documents/ Unclear	15	Gender (11 female and 04 male)	NR
**Whiting, Caldwell and Akers **[32]	UK	Qualitative research design	15 credit academic module	3 years	1st and 3rd years	Children’s nursing, pharmacy, dietetics, diagnostic radiography, radiotherapy, foundation paramedics, paramedic science, physiotherapy, and social work	Focus groups/ Thematic approach	Unclear	NR	NR
**Walker, Cross and Barnett **[35]	Australia	Mixed methods study	Rural clinical learning environments	NR	1st to 4th years	Medicine, nursing, midwifery, and allied healthcare	Open-ended survey questions, interviews and focus groups/ Unclear	60	Gender (10 male and 50 female)	NR
**O’Neill e Wyness **[33]	Canada	Qualitative research design	Disciplinary and interprofessional content and practices focused on a specific disease	NR	NR	Medicine, pharmaceutical sciences, nursing, and social work	Focus group/ Unclear	14	Gender (3 male and 20 female)	20 to 44 years
**Mu et al. **[29]	USA	Mixed methods study	Consortium for Rural, Interprofessional Training” included didactic and clinical components	3 years	NR	Occupational therapy, physical therapy, and pharmacy	IEPS/Reflection journals, experience debriefings, pre-post observation summaries, and on-site summaries /constant comparative method	111	Gender (40 male and 71 female)	18 to 46 years

*Abbreviations*: countries: *USA* United States of America, *UK* United Kingdom; instruments: *IEPS* Interdisciplinary Education Perception Scale; results*: NR* Not reported

### Participants

The graduation year of the students who participated in the IPE activities showed a wide variation, including individuals from the first to the fifth year. However, four studies entered students who were in their first year of graduation. The demographic characteristics of students were little explored, and the variable gender was considered in eight studies and age in only five (Table 2).

### Quality assessment of studies

The results obtained in the assessment of the quality of the studies are shown in Table 3. Only one article [31] had the maximum score for the CASP instrument. However, it is emphasized that most of them had a high-quality score. One study [38] obtained a score of zero in one of the three questions considered essential and, consequently, was excluded from the analysis, as it did not meet the minimum quality criteria, as recommended by the instrument.

**Table 3.**
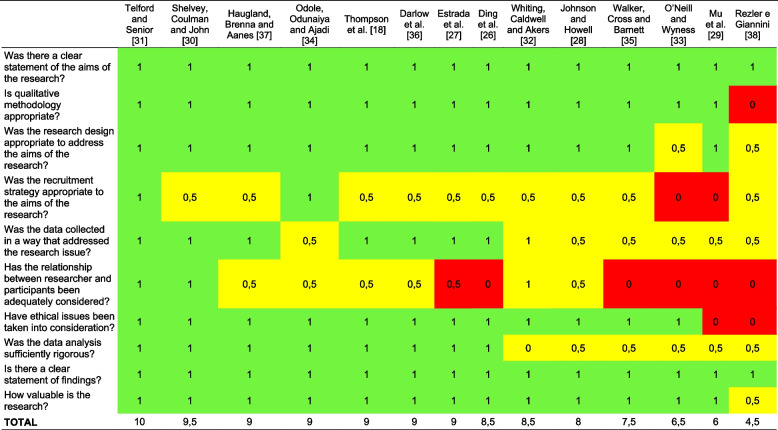
Score of the evaluated papers item by item for Critical Appraisal Skill Programme (CASP). Curitiba, 2021

The criteria of clarity of objectives, adequacy of methodology and clear assertion of research findings were met in all studies. The other CASP items, fully attended (score equal to 1), were: research design [18, 26–32, 34–37]; proper recruitment strategy [31, 34]; data collection [18, 26, 27, 30–32, 36, 37]; data analysis [18, 26, 27, 30, 31, 34, 36, 37] (Table 3). On the other hand, the research design was considered only partially appropriate to meet the objectives, in one of the studies [33], due to the long time elapsed for the beginning of the interviews. The sample recruitment strategy was considered inappropriate in two [29, 33] studies, due to the absence of a description of how the recruitment of students was carried out. The relationship between researcher and participants, during the question formulation process and the data collection stage, was inappropriate in four studies [26, 29, 33, 35] and partially appropriate in six studies [18, 27, 28, 34, 36, 37].

### Synthesis

The line-by-line coding allowed the development of a total of 72 initial codes – 51 of which were identified as potentialities and 21 as limitations for the IPE. Subsequently, a hierarchical tree structure was created to capture the meaning of the initial codes, generating a total of 16 descriptive themes. After successive discussions, five analytical themes emerged, being evidenced by 16 interpretive codes as potentialities and 16 as limitations to the IPE. A diagram (Fig. 3) was drawn up to illustrate the frequency and relevance of these interpretive codes [24].

The three analytical themes identified as potentialities were: learning from and about each other; the value of education and interprofessional practice; patient-centred health care (circles – Fig. 3). The two analytical themes, classified as IPE limiting, were named: IPE-related barriers; and difficulties related to teaching methodologies (squares – Fig. 3).

## Potentialities

### Learning from each other and about them

IPE provided students with an opportunity to learn “about, with and with each other” to improve collaboration and quality of care. The interaction with people from other courses and professions was positive and contributed to the students’ understanding, in a deeper way, of the roles and responsibilities of their peers [18, 27, 29, 30, 32–34, 36, 37]. In addition, it provided insights on how they will be able to help each other in their professional future, in a learning process that is also intersubjective:

“I thought it was good to meet other professionals and learn about where they fit in the healthcare system and how we will be integrated with them […]” [32].

“Was beneficial for her career as well as mine and learning about our different professions” [27].

The experience also favoured interprofessional collaboration [18, 28, 29, 34, 36, 37], in which the students learned to share their knowledge and skills with each member of the group, to achieve a common goal:

“I began to view collaboration differently. I learned how to use the other professions in the IPE-group collaboration and contact them when I needed it” [37].

*“I have realized the importance of admitting the limitations of one’s scope of practice and using all members of the [interprofessional collaboration] […]”* [18].

The IPE activity acted as a facilitator for interprofessional communication [18, 26, 28, 33, 34], increasing the motivation, frequency, and quality of communication between students from different courses. The dialogue was based on mutual respect for the roles and responsibilities of each profession, which helped to overcome problems related to professional limits:

“There was open communication and respect for each other’s roles […]” [18].

“I have learned […] how to communicate with all the members of the team, like the nursing staff […]” [26].

Another important finding was linked to the reduction of stereotypes [27–29, 33] about other professional categories. Ignorance of the roles and responsibilities of other professions can favour the establishment of negative stereotypes. However, the opportunity to learn from each other surprised the students, when they realized that their ‘prejudices’ about other professions were in fact incoherent:

“[…] it is difficult to break misconceptions [about other professions] when you are not really clear about what their role is. It is not until you actually work with them that you know what they do […]” [33].

“I think it has helped to erase a lot of biases I didn’t know I had” [28].

The synthesis also showed that students appreciate opportunities for interaction with other professions [18, 27, 33]:

*“Having a personal relationship with other providers makes it easier to know exactly what services they feel comfortable providing, and makes open and transparent communication easier to achieve”* [18].

This interaction, provided by the IPE, was considered essential to reduce the hierarchies present in health systems [33, 34]:

*“[…] The hierarchical nature of the health care system is a problem but if we learn together, we will be able to see what each person has to contribute and communicate effectively”* [34].

There was also increased trust [28, 33] among the team members:

“a lot of times they would just pop into the patient room [and ask] hey guys, are you sure you want to do this or we are out of this and give us suggestions” [28].

### The value of education and interprofessional practice

The opportunity to interact with people from other professions promoted an improvement in attitudes toward other professions [26–29, 32, 33, 36], evidenced by increased trust, respect, and admiration among peers. Also, the interaction provided a broader understanding of the strengths and weaknesses of each profession and how to utilize them:

“[…] there are strengths and weaknesses to each profession […] you are relying on each other heavily to find those out and utilize them” [28].

“[…] taught me to quiet my mouth and realize that other people have important things that they can say too” [27].

The work experience, in classrooms and in clinical environments, promoted some reflections on how the activity could impact your professional future [28, 30, 32, 33]:

“Yeah, we formed relationships for future practice, when an oral presentation’s involved, and we were preparing for future holistic care” [36].

“[…] being able to observe different professions working together as a team motivated me to want to do the same in my professional career […]” [33].

There were reports on how the IPE activity enabled professional and personal growth [26, 28, 36, 37]: one student reported that the experience would help her *“not shy away from interprofessional interactions in the future”* [28]. Furthermore, the activity helped to value the different points of view and share the responsibilities involved in a work environment:

“[…] My competence became inherently valuable, but some time elapsed before I realized it. I noticed that I became more receptive to the competence of others and was more likely to see things from other people’s perspectives” [37].

Dialogue and the opportunity to establish social connections played a key role in producing mutual respect among students [18, 30, 33, 36]:

“[…] it helps to see where they’re coming from when you actually go to work … there is a different respect level, knowing where they are at […]” [33].

The experience enabled the establishment of a professional identity [27, 32, 35] of its own:

“I think a lot of people are quite confused about the different kind of nursing you can do. [But] we could all express exactly what our role is […]” [32].

Students appreciate opportunities for informal interactions [28, 29, 33]. Frequent contact with other students, outside the discipline, favoured the interprofessional relationship of the participants:

“[…] a lot of it happens … in the halls and drinking coffee […] it was really a good idea that they […] forced us at the beginning [to work in student interprofessional teams] [33].

“[…] you work together and then you are together at night” [28].

### Patient-centred health care

The students emphasized the value of collaborative work in establishing patient-centred care [26, 29, 32], to provide quality-based treatment. Thus, the students expressed that the interprofessional team can provide an *“optimal patients care”.* And in that way, it can “*help patients get the best treatment they can”.* In addition to being considered *“vital for successful rehabilitation of every patient”* [29].

Furthermore, the opportunity to experience the IPE allowed students to reflect on the importance of humanizing care [27, 28, 32] and the value of promoting holistic patient care: *“I think it makes you think of the family as a whole rather than just the patient”* [32]. For others, the experience helped *“learn how to speak to people and just being company to them”* [27]. In addition, the presence of a representative from each course/profession allowed the team to *“target different things”* and *“to improve the outcome of the patient”* [28].

## Limitations

### Barriers related to IPE

The interaction between students was marked by some challenges [28, 33–35, 37]. Among them, the time spending to make explanations to the group: *“A lot of time was [spent] explaining your point of view to other people […]”* [33]; and the difficulty in finding similar skills to promote greater patient care: *“[The difficulty is] finding common ground, that is, similar clinical skill sets, in order to achieve a benefit”* [35]. Furthermore, they did not know how to act in the face of differences of opinion and how to ask their peers for help. However, the experience allowed them to learn to deal with these problems: *“we had to figure out how to handle it […]”* [28].

The students noted in their placements that not all people actually know what they really do, highlighting the misunderstanding of roles and responsibilities [27, 30, 32, 34] between different professions: *“I think a lot of people are quite confused about the different kind of nursing you can do”* [32]. However, even after the activities were completed, there were reports of doubts about the roles and responsibilities of other health professionals who had contact: *“I still don’t understand all different levels of being a nurse. I asked and I learned from the nursing student. I am still a bit confused”* [27].

The challenges motivated by disagreements in teamwork created an environment of dissatisfaction [27, 33, 36] among some students. Further, the lack of recognition and collaboration contributed to establishing a sometimes-conflicting environment:

“[…] I didn’t feel comfortable in the group […] I was just sort of silenced. And I found out when I did speak about things, it wasn’t really acknowledged […]” [33].

“[…] It is just frustrating when there is paperwork due from the whole group, yet one person has to do it” [27].

There were times when students experienced a feeling of insecurity [26, 27, 33], with internal questions during practical activities:

“I think there were times where maybe I would like to ask a question that if it was just med students, interns, residents and attending, maybe I would have brought up that question and we could have discussed a little bit further” [26].

Another limiter aspect was related to the loss of opportunities [35, 36], that is, circumstances in which students failed to take advantage of interprofessional learning occasions to prioritize other activities: *“In the hospital there is some downtime, but we tend to use it for catching up on study […] rather than exploring other people’s roles”* [35].

Also, there were situations in which students showed resistance to the IPE [26, 34]. The feeling of loss of knowledge of their specific professional area, the lack of receptiveness to working with other professionals, and the feeling of “overprotection” of their professional field, were factors that negatively impacted the predisposition to IPE:

“You don’t have that time to sit and discuss things because you are with the whole team. […] I think that is a challenge as a med student and because you know the whole reason why we come is to try to learn how to manage these illnesses and things, you know, not so much how to work with the caseworker. It is good to learn that, [but] right now that is not necessarily what our goal is” [26].

### Difficulties related to teaching methodologies

Students reported that it would be important to present some concise and relevant information before the IPE [30, 35] activity: *“Providing an understanding of the roles and capabilities in [of] professions outside of medicine would allow for a much higher degree of respect towards these disciplines and enhance the effectiveness of teams, thus improving patient care”* [35].

The preparation prior to activity was also suggested, to facilitate the understanding of the activities to be carried out: *“I also think there should have been a preparation task we had to do so an e-learning module and something interactive, a quiz maybe. I think by doing a bit of work in the topic area before I would have got more out the session then”* [30].

The absence of assessment [36] was also considered a barrier to better absorption of IPE. Consequently, students who were not evaluated felt that the presence of a grade/concept could influence them to put more effort into their activities. Furthermore, the excess of theoretical classes was considered unproductive [34]. Choosing an inappropriate location for theoretical and practical classes [30] an affect the interaction and enjoyment of these activities.

The excess of students in the same class was also considered a negative factor. The reduction of class size would promote greater interaction with peers: “*I think it obviously would have made the organization a lot easier cos there would be a lot less people”* [30].

Regarding the workload, the students suggested that it could be expanded and distributed recurrently in the curriculum: *“if it’s a one off thing people just think well it’s not that important. If it was something that built in systematically then you think it’s important”* [30]. In addition, in one case, the lack of interaction with students from other professions generated frustration: *“Well, IPE in the College of Medicine to me is zero, because we virtually don’t do anything together”* [34].

## Discussion

The main findings of this review can contribute to a greater understanding of students’ perceptions about their experiences and improve the implementation of the IPE. The emphasis of the review was directed to the evidence on the potentialities and limitations of the IPE, in front of the teaching–learning of undergraduate students in the health area. Among the potentialities, three analytical themes were identified: learning from each other and about them; the value of education and interprofessional practice; patient-centred health care. On the other hand, some limitations that can hinder student engagement were also pointed out, such as: the barriers related to IPE; and the difficulties related to teaching methodologies.

IPE provided students with the opportunity to learn from and about each other. This interaction with people from other courses enabled them to better understand the roles and responsibilities [18, 27, 29, 32–34, 36, 37] of each profession, developing appreciation and respect to each other’s roles. The findings of this review are consistent with other studies [39, 40], which emphasize understanding the scopes of practice in each course and profession, in view of a successful interprofessional practice. On the other hand, misunderstanding is part of the elements already present in traditional curricula, which collaborate to fragmented health care [41]. Thus, it is desirable that this teaching–learning approach be incorporated, assuming as plausible its positive impact on the provision of health services.

Another item that stood out was the improvement in attitudes towards other professions [26–29, 32, 33, 36]. Corroborating these findings, other studies also pointed out this ability of the IPE to foster positive attitudes towards other professions [42, 43]. Kenaszchuk et al. [44] also emphasized, which skills can be improved: the perception of competence and autonomy of other professionals, communication, teamwork, and attitudes towards interprofessional learning. However, Hall [45] emphasizes that, to achieve these goals, it is essential that there is an early immersion of students in this teaching approach. Therefore, the introduction of IPE curricula in HEIs can help to reduce negative stereotypes, favouring teamwork and collaborative practice, after the insertion of these professionals into health systems.

The opportunity to experience interprofessional collaboration [18, 28, 29, 34, 36, 37] in practice allowed students to understand that all professions have unique skills, but that they can be integrated and complemented. Reeves [8] also observed positive changes of students in relation to interprofessional collaboration, evidenced by the development of interprofessional skills and mutual respect. Therefore, IPE can induce students to acquire skills, attitudes, and knowledge, preparing professionals with the necessary skills to act in the face of new global challenges.

Despite the benefits mentioned above, negative experiences were also reported. Students identified, for example, tensions and challenges in interprofessional interaction [28, 33–35, 37] and dissatisfaction with teamwork [27, 33, 36]. To solve these problems, Reeves [8] suggests that learning mediators exhibit certain characteristics, such as: experience in IPE; knowledge of interactive learning methods and group dynamics; and flexibility to sustain an environment of mutual respect. Thus, it is essential that IPE activities are promoted by professionals with experience and skills to deal with conflict situations.

Furthermore, it was observed in some reports that students also revealed a misunderstanding of professional roles [27, 30, 32] even after the IPE activities. Accordingly, some studies explain that this lack of understanding can be caused by several factors, such as negative stereotypes, prejudices, and intergroup discrimination [15, 46, 47], yet it is mainly impacted by the learning context [15]. Thus, to neutralize this barrier, it is essential that mediators discuss the different roles and responsibilities of each profession. In addition, it is important that these students are included in interprofessional curricula in their first year of graduation, through “icebreaker” activities, to reduce possible tensions in their future practices.

The feeling of insecurity [26, 27, 33] was also evidenced as a limitation to IPE. Kyprianidou et al. [48], in a study that aimed to explore the impact of forming heterogeneous groups on teamwork, revealed that students feel insecure in collaborating with unknown people. Nevertheless, those who left their comfort zone reported benefits of heterogeneity and pluralism in their reasoning. To reduce this feeling, it is suggested that students have prior contact with colleagues from other professions in an “icebreaker” activity, to reduce possible tensions during the activities.

The synthesis of the findings involved some limitations, which were conditioned by intrinsic factors of each institution. Among them, the following stand out: learning activities (classroom and clinical environment), duration, professions involved, academic year, number of participants, study design, data collection, among other factors. It is noteworthy that most of the studies involved punctual actions [26, 28, 30, 31, 36, 37], a fact that can influence the perception of students about this teaching approach, due to the limited time of practice and its episodic nature, not systematic. Thus, it is essential to develop studies that assess the long-term impact of IPE to demonstrate the effectiveness and efficiency of this teaching model. In addition, information such as: age, gender, and previous experience in IPE are factors that can influence students’ perception of this teaching–learning modality [49–51]. Thus, they should be considered in studies that evaluate IPE curricula.

The quality assessment of the included works was performed to provide a transparent and robust assessment of the studies that make up the synthesis. Most studies achieved a high score on the CASP instrument, but only one article [31] had a maximum score. The item “clarity of objectives”, “adequacy of the methodology” and “clear presentation of the findings” was met in full in all studies. In contrast, there was little attention to the “sample recruitment strategy” [29, 33]. Adequate recruitment is crucial, as losses to follow-up can undermine the validity of the results obtained [52]. Likewise, four studies [26, 29, 33, 35] disregarded the relationship between researcher and participants during the process of formulating the questions and collecting data. The lack of critical examination of this relationship can bring potential risks of bias, with implications for research design. In short, the absence of a clear statement of these items can limit the conclusions from being extrapolated to other contexts.

The students’ training process involves the development of skills and competences that contribute to the resizing of clinical practice. However, changing the institutional culture requires deepening specific knowledge of the profession and its integration with the various disciplines and professions, in addition to the creation of new teaching methodologies and strategies, and teacher training [53]. In this way, curricular integration can stimulate students to produce extramural knowledge that will be essential for professional practice.

## Conclusion

The synthesis of the studies revealed the positive potential of this teaching approach for the formation of students, with interprofessional skills and competences necessary for the future provision of high-quality health care. IPE has an emphasis on interprofessional interaction as the teaching and learning process, this possibility of interaction with students from different areas is a differential of IPE compared to other teaching methodologies. Thus, we can observe the courses that most use this teaching approach are Medicine and Nursing. Other professions, despite also being part of the health team, are trained mainly in the uni-professional teaching model. Despite the existence of a diversity of teaching and learning methods, standardization becomes favorable for the purpose of evaluating and monitoring the results of the IPE. However, it is important to consider the context of each Higher Education Institution, such as: professions involved, academic year, number of participants, etc., to achieve the central objective of the IPE: “occurs when two or more professions learn with, about, and from each other to enable effective collaboration and improve health outcomes”. Therefore, the perceptions and depositions given by students can help Higher Education Institutions to institute or improve their IPE approaches.

## Supplementary Information

**Additional file 1: Supplement 1.** Electronic database and search strategy.

**Additional file 2: Supplement 2.** Questions regarding the Critical Appraisal Skills Program (CASP).

## Data Availability

All data generated or analysed during this study are included in this published article [and its supplementary information files].
