# Supplementary material for: Potentialities and limitations of Interprofessional Education during graduation: a systematic review and thematic synthesis of qualitative studies
Source: BMC Med Educ. 2023 Apr 12;23:236. doi: 10.1186/s12909-023-04211-6 (PMC10099638; doi:10.1186/s12909-023-04211-6)
Supplement: Supplementary file 1 — Additional file 1: Supplement 1. Electronic database and search strategy. [file 12909_2023_4211_MOESM1_ESM.docx]

Supplement 1. Electronic database and search strategy

| PubMed | Search (((((((Students[MeSH Major Topic]) OR Students[Title/Abstract]) OR Graduat*[Title/Abstract]) OR "College Students"[Title/Abstract]) OR "Higher Education"[Title/Abstract])) AND ((((((("Interdisciplinary Communication"[MeSH Major Topic]) OR "Interprofessional Relations"[Title/Abstract]) OR "Interprofessional Collaboration"[Title/Abstract]) OR "Interprofessional Education"[Title/Abstract]) OR "Collaborative Practice"[Title/Abstract]) OR "Collaborative Learning"[Title/Abstract]) OR Teamwork[Title/Abstract] OR “Interprofessional Learning”[Title/Abstract]))) | 4.067 |
| --- | --- | --- |
| Cochrane Library | #1 MeSH descriptor: [Students] this term only OR #2 (Student*):ti,ab,kw OR #3 (Graduat*):ti,ab,kw OR #4 ("Education, Professional"):ti,ab,kw OR #5 ("College Students"):ti,ab,kw OR #6 ("Higher Education"):ti,ab,kw AND #8 MeSH descriptor: [Interdisciplinary Communication] explode all trees OR #9 ("Interprofessional Education"):ti,ab,kw OR #10 (“Interprofessional Relations”):ti,ab,kw OR #11 (“Interprofessional Teamwork”):ti,ab,kw OR #12 (“Interprofessional Practice”):ti,ab,kw OR #13 (“Interprofessional Collaboration”):ti,ab,kw OR #14 (“Collaborative Practice”):ti,ab,kw OR #15 ("Collaborative Learning"):ti,ab,kw OR #16 (Teamwork):ti,ab,kw OR #17 (“Interprofessional Learning”):ti,ab,kw | 246 |
| Scielo | (subject:Student) OR (subject:Graduate) OR (subject:"Education, Professional" ) OR (subject:"College Students" ) OR (subject:"Higher Education" ) OR (subject:Estudantes) OR (subject:Estudante) OR (subject:Universitários ) OR (subject:Universitário) OR (subject:Alunos) OR (subject:Estudiantes) OR (subject: Estudiante) OR (subject:"Estudiante Universitário") OR (subject:"Estudiantes Universitários") AND (subject:"Interdisciplinary Communication") OR (subject:"Interprofessional Education") OR (subject:"Interprofessional Relations") OR (subject:"Interprofessional Practice") OR (subject:"Interprofessional Collaboration") OR (subject:"Collaborative Practice") OR (subject:"Collaborative Learning") OR (subject:Teamwork) OR (subject:"Interprofessional Learning") OR (subject:"Educação Interprofissional") OR (subject:"Trabalho em Equipe") OR (subject:"Relações Interprofissionais") OR (subject:"Relação Interprofissional") OR (subject:"Práticas Colaborativas") OR (subject:"Educación Interprofesional") OR (subject:"Relaciones Interprofesionales") OR (subject:"Aprendizaje Colaborativo") OR (subject:"Trabajo en Equipo") OR (subject:"Colaboración Interprofesional") OR (subject:"Equipos Interprofesionales") | 2.645 |
| LILACS | (tw:(Student)) OR (tw:(Graduate)) OR (tw:("Education, Professional")) OR (tw:("College Students")) OR (tw:("Higher Education")) OR (tw:(Estudantes)) OR (tw:(Estudante)) OR (tw:(Universitários)) OR (tw:(Universitário)) OR (tw:(Alunos)) OR (tw:(Estudiantes)) OR (tw:(Estudiante)) OR (tw:("Estudiante Universitário")) OR (tw:("Estudiantes Universitários")) AND (tw:("Interdisciplinary Communication")) OR (tw:("Interprofessional Education")) OR (tw:("Interprofessional Relations")) OR (tw:("Interprofessional Practice")) OR (tw:("Interprofessional Collaboration")) OR (tw:("Collaborative Practice")) OR (tw:("Collaborative Learning")) OR (tw:(Teamwork)) OR (tw:("Interprofessional Learning")) OR (tw:("Educação Interprofissional")) OR (tw:("Trabalho em Equipe")) OR (tw:("Relações Interprofissionais")) OR (tw:("Relação Interprofissional")) OR (tw:("Práticas Colaborativas")) OR (tw:("Educación Interprofesional")) OR (tw:("Relaciones Interprofesionales")) OR (tw:("Aprendizaje Colaborativo")) OR (tw:("Trabajo en Equipo")) OR (tw:("Colaboración Interprofesional")) OR (tw:("Equipos Interprofesionales")) | 1.799 |
