# Supplementary material for: Potentialities and limitations of Interprofessional Education during graduation: a systematic review and thematic synthesis of qualitative studies
Source: BMC Med Educ. 2023 Apr 12;23:236. doi: 10.1186/s12909-023-04211-6 (PMC10099638; doi:10.1186/s12909-023-04211-6)
Supplement: Supplementary file 2 — Additional file 2: Supplement 2. Questions regarding the Critical Appraisal Skills Program (CASP). [file 12909_2023_4211_MOESM2_ESM.docx]

Supplement 2. Questions regarding the Critical Appraisal Skills Program (CASP).

| 1. Was there a clear statement of the aims of the research? |
| --- |
| 1. Is a qualitative methodology appropriate? |
| 1. Was the research design appropriate to address the aims of the research? |
| 1. Was the recruitment strategy appropriate to the aims of the research? |
| 1. Was the data collected in a way that addressed the research issue? |
| 1. Has the relationship between researcher and participants been adequately considered? |
| 1. Have ethical issues been taken into consideration? |
| 1. Was the data analysis sufficiently rigorous? |
| 1. Is there a clear statement of findings? 2. How valuable is the research? |

Source: Critical Appraisal Skills Programme [23]
